# Early *Versus* Routine Oral Glucose Tolerance Test in Women With Intermediate Hyperglycemia at First Prenatal Visit: A Retrospective Cohort Study in China

**DOI:** 10.3389/fendo.2021.743170

**Published:** 2021-12-15

**Authors:** Yunzhen Ye, Kaizhou Qin, Yu Xiong, Jiangnan Wu, Qiongjie Zhou, Xirong Xiao, Xiaotian Li

**Affiliations:** ^1^ Department of Obstetrics, Obstetrics and Gynecology Hospital of Fudan University, Fudan University, Shanghai, China; ^2^ Laboratory of Female Reproduction and Endocrinology, Obstetrics and Gynecology Hospital of Fudan University, Fudan University, Shanghai, China

**Keywords:** oral glucose tolerance test, intermediate hyperglycemia, larger for gestational age, gestational diabetes (GDM), adverse pregnancy outcomes

## Abstract

**Background and Objectives:**

Intermediate hyperglycemia in the first half of pregnancy, defined as a fasting plasma glucose level between 5.1- 6.9 mM, increases the risk of gestational diabetes mellitus, but clinical evidence for further management is lacking. We aim to evaluate the effectiveness of an early oral glucose tolerance test (OGTT) followed by the identification of intermediate hyperglycemia on pregnancy outcomes in real world setting.

**Subjects and Methods:**

A retrospective cohort study was conducted at the Obstetrics and Gynecology Hospital, Shanghai, China, between 2013 and 2017. Women with intermediate hyperglycemia at the first prenatal visit were identified and underwent an immediate (within one week) or a routine OGTT (24-28 gw) according to their wishes and received nutrition and exercise advice. Women diagnosed of gestational diabetes (GDM) were managed by standard interventions. Primary outcome was larger for gestational age (LGA). Secondary outcomes were primary cesarean delivery, preterm birth, shoulder dystocia or forceps delivery, preeclampsia, neonatal hypoglycemia, hyperbilirubinemia, and low Apgar score. Logistic regressions with or without a further propensity score-matched analysis were performed.

**Results:**

Among 42406 women involved, 1104 (2.6%) with intermediate hyperglycemia at the first prenatal visit were identified, of whom 176 (15.9%) underwent an early OGTT and 741 (67.1%) received a routine OGTT. Logistic regression showed that an early OGTT was not significantly associated with an altered risk of LGA (adjusted OR 1.13, 95% CI 0.73-1.75) but was related to an increased odds for neonatal hyperbilirubinemia (adjusted OR 2.89; 95% CI 1.55-5.37). No significant associations were observed for other secondary outcomes. These trends remained consistent in propensity score-matched models.

**Conclusions:**

Our data from a real-world setting did not support that an early OGTT among women with intermediate hyperglycemia at the first prenatal visit improved pregnancy outcomes.

## Introduction

Gestational diabetes mellitus (GDM) is a common complication during pregnancy, affecting 15% of pregnancies worldwide (1). Intermediate hyperglycemia is not life-threatening for mothers by itself, but it harms the fetus, leading to short-term and long-term complications, including larger for gestational age (LGA), preterm birth and later-life metabolic disorder (1–3). Screening and identifying GDM improve maternal and fetal outcomes. Based on available evidence, the recommendation of the International Association of Diabetes and Pregnancy Study Group (IAPDSG) for universal screening for GDM after 24 weeks of gestation by a 75 g oral glucose tolerance test (OGTT) is accepted without doubt or controversy (2, 4). In contrast, before 24 gestational weeks (gw), there is consensus to carry out risk factor-based screening at the first prenatal visit (4).

There is evidence from randomized trials that in women with risk factors for GDM, early identification of GDM by the 75 g-OGTT before 24 gw helps to reduce the large for gestational age (LGA) rate (5). Intermediate hyperglycemia at the first prenatal visit, defined as a fasting plasma glucose (FPG) level in the 5.1 to 6.9 mM range (6), increases the risk for GDM, diagnosed after 24 gw, by approximately ten times, contributing to a higher frequency of LGA and primary cesarean section (7). However, clinical evidence regarding how to manage these high-risk women is lacking. The recommendation of IAPDSG of classifying an FPG≥5.1 mM as GDM is more pragmatic than evidence based and is thereby controversial (4, 6). Data involving the Chinese population showed that an FPG≥5.1 mM at the first prenatal visit could not be the proper criterion for the diagnosis of GDM, as it obtained low sensitivity and specificity (8). Thus, women with intermediate hyperglycemia at the first prenatal visit should be regarded as being at high risk for GDM, and an early OGTT may help to improve pregnancy outcomes. However, evidence regarding the effects of an early OGTT before 24 gw among mothers with intermediate hyperglycemia in a real-world setting is lacking.

The irrationality of using a single value of FPG≥5.1 mM before 24 gw to diagnose GDM may be associated with the unrepeatability of FPG during pregnancy (9–11). A further OGTT before 24 gw among those with intermediate hyperglycemia may help to early identify insulin resistance and improve pregnancy outcomes. Thus, a retrospective cohort study was designed to test the hypothesis that among women with intermediate hyperglycemia at the first prenatal visit, an immediate OGTT (early OGTT), compared with a routine OGTT at 24-28 gw, would improve pregnancy outcomes by conferring early identification and management of GDM. The primary outcome was LGA, and the secondary outcomes were primary cesarean delivery, preterm birth, shoulder dystocia or forceps delivery, preeclampsia, neonatal hypoglycemia, hyperbilirubinemia, and low Apgar score. This study will provide evidence for the necessity to perform an early OGTT among mothers with intermediate hyperglycemia at the first prenatal visit.

## Methods

### Study Design and Participants

**Figure 1:**
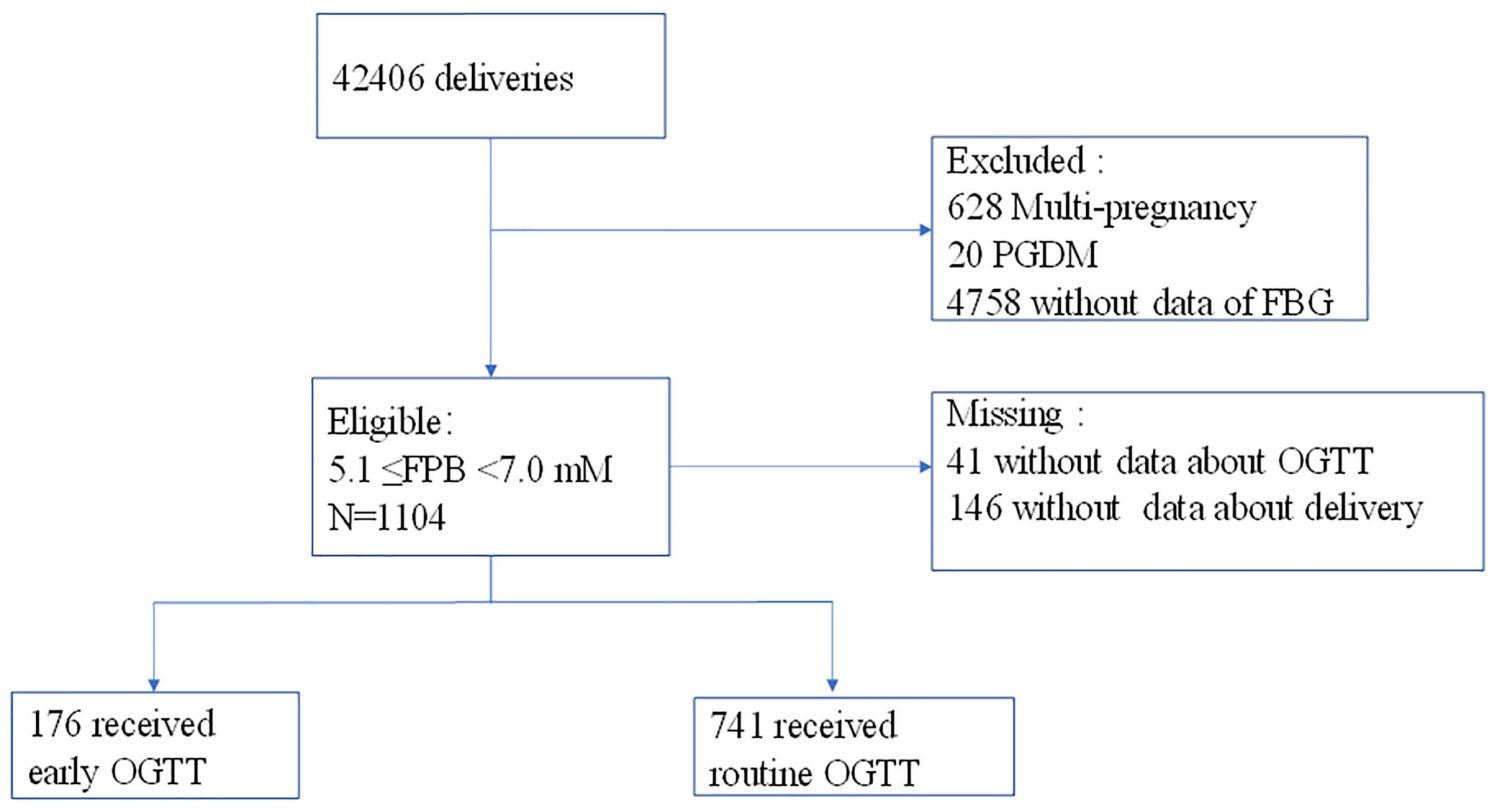
Process of participant selection and recruitment of the study. A total of 41 participants who had no screening data and 146 women who had no delivery data were excluded.

This was a retrospective cohort study comparing pregnancy outcomes associated with an early OGTT (≤21 weeks of gestation) and a routine OGTT (24-28 gw) among women with intermediate hyperglycemia (5.1≤PBG<7.0 mM) at the first prenatal visit (≤20 weeks of gestation). Women who had records of Down’s screening (≤20 weeks of gestation) and later delivered at the Obstetrics and Gynecology Hospital of Fudan University, Shanghai, China, from 2013.01.01 to 2017.12.31 were involved. The maternal age of the participants was restricted to 18-40 years old, and those who had pregestational diabetes (PGDM) (N=20), multiple pregnancies (N=628) and no FPG data were excluded. Finally, 37700 women were involved, and 1104 (2.93%) of them with intermediate hyperglycemia (5.1 ≤FPG<7.0 mmol/L) at the first prenatal visit were identified; of these women, 41 women without data of further screening and 146 women without information about delivery were excluded. Thus, 917 women with intermediate hyperglycemia were included in the final analysis (**Figure 1**). On identification of intermediate hyperglycemia at the first prenatal visit, women were educated about the risk of gestational diabetes and chose to undergo an early OGTT (within 1 week) or a routine OGTT (24-28 gw) by the 75-g OGTT according to their own wishes. GDM was diagnosed based on the International Association of Diabetes and Pregnancy Study Group (IADPSG, 2010) criteria. On diagnosis of GDM, patients were transferred to nutrition clinics, provided diet and exercise advice, underwent home blood glucose monitoring and insulin therapy if required, and terminated pregnancy according to the IADPSG guidelines. Additionally, a secondary OGTT at 24-28 gw was advised if the women were not diagnosed with GDM at the early OGTT. Otherwise, if the women chose to receive a routine OGTT at 24-28 gw rather than an early OGTT, they could also receive advice to balance diet and exercise at the first prenatal visit. This study was approved by the Ethics Committee of the Obstetrics and Gynecology Hospital.

### Diagnosis of PGDM and GDM

GDM was diagnosed according to the IADPSG (2010) criteria by the 75-g OGTT after at least 3 days of maintaining a normal diet with carbohydrates within 250-300 g and normal activities. Briefly, a diagnosis of GDM was made if the maternal blood glucose level met or exceeded at least one of the following thresholds: FPG, 5.1 mM; 1-h plasma glucose, 10.0 mM; and 2-h plasma glucose, 8.5 mM. PGDM was defined as at least one of the abnormal values on the OGTT: FPG ≥7.0 mM or 2-h plasma glucose≥11.1 mM; or glycated hemoglobin≥6.5%; random glucose level≥11.1 mM with overt syndrome of diabetes.

### Data Collection

Demographic, biochemical, antenatal, intrapartum, and postpartum clinical outcomes of each mother and infant were extracted from the mother’s electronic health record by a special commissioner. Additional clinical data of the infants were extracted from neonatal health records. All gestational ages were calculated based on antenatal ultrasound scans to establish the agreed estimated delivery data. Maternal body mass index (BMI) was recorded at the Downs’ screening at 16-20 gw.

### Outcomes

This study was conducted to estimate whether an early OGTT in women with intermediate hyperglycemia at the first prenatal visit could improve pregnancy outcomes compared with routine screening at 24-28 gw. Thus, primary and secondary pregnancy outcomes were defined based on the hyperglycemia and Adverse Pregnancy Outcomes Study (2). The primary outcome was LGA, while the secondary outcomes included primary cesarean section, preterm birth, preeclampsia, forceps delivery or shoulder dystocia, neonatal hypoglycemia, and hyperbilirubinemia.

LGA and SGA were defined as a newborn weight higher than the 90^th^ percentile or less than then 10^th^ percentile respectively, which was based on INTERGROWTH-21^st^ (girls and boys). Neonatal hypoglycemia was defined as a capillary blood glucose level lower than 2.6 mmol/L at delivery, and only infants with LGA, SGA, or macrosomia (≥4000 g) born to mothers with GDM were examined for blood glucose at delivery. Preterm birth referred to termination of pregnancy at or after 28 gw and before 37 gw, regardless of whether it was spontaneous or iatrogenic preterm delivery. The diagnostic criteria for preeclampsia were new onset of both hypertension (systolic blood pressure of 140 mmHg or higher, diastolic blood pressure of 90 mmHg or higher, or both) and proteinuria (≥300 mg of protein per 24-hour urine collection, 1+protein or greater on dipstick urine analysis, or a ratio of protein to creatine of spot urine≥0.3) after 20 gw.

### Analysis

Statistical analysis was carried out on an intention-to-treat basis. Analysis was performed before and after propensity score matching. Continuous variables are presented as the mean ± standard deviation (SD), and categorical variables are shown as n (%). Independent t tests or Mann-Whitey U tests were used to compare continuous variables if they followed a normal or nonnormal distribution, respectively. The chi-square test followed by Bonferroni corrections was applied to compare categorical variables. The normal distribution of continuous variables was evaluated using the Kolmogorov-Smirnov test. Propensity score matching was used to identify a cohort of patients with similar baseline characteristics between groups. Matching was performed with the use of a 1:2 matching with a caliper width equal to 0.02. The covariables involved in propensity score matching were maternal age, BMI and HbA1c level.

Multivariable logistic regression models for pregnancy outcomes were developed. The selection of the covariates to be included in the models was based on findings in previous scientific literature (12, 13), and variables of maternal age, BMI and HbA1c level were retained in the model irrespective of statistical significance because all these covariates had a theoretical association with the outcomes. We performed a sensitivity analysis in which we excluded participants without a second OGTT in the early screening group because some of these women may have had GDM but received no related management. Furthermore, a *post hoc* analysis was conducted by sub-analysis of maternal age, BMI, HbA1c level, number of parturitions and fetal sex. The cutoff of maternal age was 29 according to our previous finding of an optimized cutoff maternal age for GDM (14). The cutoff of maternal BMI was made at 25 according to the definition of overweight or not. The cutoff of the level of HbAlc was 5.2% according to 75% HbAlc in the recent population.

A total of 16.9% of the data for the OGTT or delivery outcomes were missing, and we made no attempts to impute missing data because we had no information about whether these data were missing at random. A two-sided p-value of <0.05 was considered statistically significant. All analyses were performed using SPSS 23.0.

## Results

### Baseline Characteristics

Between January 2013 and December 2017, a total of 1104 women with intermediate hyperglycemia at the first prenatal visit were identified (**Figure 1**). The analysis included 917 eligible participants who could be evaluated, of whom 176 underwent early screening and 741 underwent routine screening (**Figure 1**). The proportion of women diagnosed with GDM was higher in women who underwent an early OGTT than in those who underwent a routine OGTT (49.4% vs 32.5%, P<0.05) (**Table 1**). The occurrence of LGA did not differ between the two groups (18.8% vs 15.9%, P>0.05) (**Table 1**).

**Table 1:** Maternal characteristics before and after Propensity-Score matching*.

	Before matching			After matching#		
	Routine OGTT (n = 741)	Early OGTT (n = 176)	P value	Routine OGTT (n = 313)	Early OGTT (n = 170)	P value
**Maternal characteristics**						
Age (years)	29.66 ± 3.19	29.38 ± 3.18	0.30	29.64 ± 3.14	29.56 ± 3.14	0.79
BMI (kg/m^2^)			0.45			0.99
<18.5	55 (7.4%)	12 (6.8%)		15 (4.8%)	12 (7.1%)	
18.5-24.9	472 (63.7%)	103 (58.5%)		214 (68.4%)	104 (61.2%)	
25-29.9	172 (23.2%)	47 (26.7%)		75 (24.0%)	45 (26.5%)	
≥30	35 (4.7%)	12 (6.8%)		9 (2.9%)	9 (5.3%)	
Primiparous	651 (87.9%)	156 (88.6%)	0.77	276 (88.2%)	152 (89.4%)	0.68
Educational level			0.86			0.99
<college	298 (40.4%)	72 (41.1%)		125 (40.2%)	68 (40.2%)	
≥college	439 (59.6%)	103 (58.9%)		186 (59.8%)	101 (59.8%)	
Smoking	13 (1.8%)	4 (2.3%)	0.65	7 (2.2%)	3 (1.8%)	0.73
Family history of DM or previous GDM	84 (11.3%)	22 (12.5%)	0.66	32 (10.2%)	21 (12.4%)	0.47
Previous macrosomia	5 (0.7%)	2 (1.1%)	0.53	3 (1.0%)	2 (1.2%)	0.82
Previous PCOS	5 (0.7%)	1 (0.6%)	0.88	4 (1.3%)	1 (0.6%)	0.47
First prenatal visit						
Gestational age	12.13 ± 3.08	10.15 ± 3.95	0.27	12.36 ± 2.87	10.05 ± 3.96	<0.01
FBG (mmol/L)	5.36 ± 0.37	5.36 ± 0.34	0.80	5.35 ± 0.36	5.35 ± 0.34	0.91
HbAlc (%)	4.98 ± 0.45	5.09 ± 0.41	0.02	5.06 ± 0.33	5.06 ± 0.31	0.97
OGTT						
Gestational age	24.94 ± 1.45	12.42 ± 4.64	<0.01	24.95 ± 1.45	12.29 ± 4.66	<0.01
0h (mmol/L)	4.73 ± 0.56	4.84 ± 0.44	<0.01	4.74 ± 0.49	4.83 ± 0.43	0.04
1h (mmol/L)	8.24 ± 1.96	8.35 ± 1.82	0.50	8.38 ± 1.90	8.30 ± 1.83	0.70
2h (mmol/L)	6.93 ± 1.58	7.06 ± 1.65	0.36	7.12 ± 1.50	6.97 ± 1.60	0.31
Total GDM	241 (32.5%)	87 (49.4%)	<0.01	120 (38.3%)	82 (48.2%)	0.04
**Newborn characteristics**						
Gestational age at delivery (gw)	39.15 ± 1.62	38.90 ± 1.64	0.08	39.13 ± 1.65	38.93 ± 1.80	0.25
Birth weight (g)	3345.87 ± 494.32	3350.80 ± 505.74	0.91	3335.30 ± 499.39	3336.88 ± 486.91	0.97
Male sex	355 (47.9%)	77 (43.8%)	0.32	157 (50.2%)	73 (42.9%)	0.13

*Data are mean ± SD or N (%).

^#^Matching was performed with the use of a 1:2 matching with a caliper width equal to 0.02. Covariables involved in propensity-score matching were maternal age, BMI and HbA1c.

Maternal age, BMI and smoking status did not differ significantly between the participants who underwent an early OGTT and those who underwent a routine OGTT. A slightly higher concentration of hemoglobin A1c (HbA1c) was observed in the participants with an early OGTT than in those with a routine OGTT (4.98% ± 045% vs 5.09% ± 0.41%, P=0.02) (**Table 1**). In terms of newborn characteristics, no significant difference was found in gestational age at delivery, birth weight or male sex proportion between the early OGTT and routine OGTT groups.

After propensity score matching, two groups of 483 well-matched cases were generated and balanced regarding baseline characteristics (**Table 1**).

### Pregnancy Outcomes Between Early OGTT and Routine OGTT

The odds ratios (ORs) for adverse pregnancy outcomes associated with an early OGTT according to logistic regression models are highlighted in **Table 2**. In models before propensity score matching, an early OGTT did not alter the odds of LGA in either unadjusted or adjusted models (**Table 2**). Neither sensitivity analysis by exclusion of cases without a second OGTT nor propensity score matching changed this trend (**Table S1** and **Table 2**). In terms of secondary outcomes, infants of mothers with early screening had a significantly increased risk of hyperbilirubinemia in both unadjusted (unadjusted OR 2.90, 95% CI 1.57-5.38) and adjusted models (adjusted OR 2.89, 95% CI 1.55-5.37) (**Table 2**). In both the sensitivity analysis and propensity score matching, statistical significance remained (adjusted OR 3.02, 95% CI 1.54-5.59; adjusted OR 3.17, 95% CI 1.45-6.90, respectively) (**Table S1** and **Table 2**). An early OGTT was related to an increased risk of forceps delivery or shoulder dystocia in the sensitivity analysis (adjusted OR 2.28, 95% CI 1.02-5.09) (**Table S1**), although this trend was not observed in the models before the sensitivity analysis (P>0.05) (**Table 2**). In all the models, no significant findings were observed for other secondary outcomes, including primary cesarean birth, SGA, preterm birth, preeclampsia, neonatal hypoglycemia, and neonatal low Apgar score (P>0.05) (**Table 2** and **Table S1**).

**Table 2:** Primary and secondary outcomes between early OGTT and routine OGTT.

	Before Propensity-Score Matching					After Propensity-Score Matching				
	Routine OGTT^#^ (n = 741)	Early OGTT^#^ (n = 176)	P value	Model 1^&^ Unadjusted OR (95%CI)	Model 2^&^ Adjusted OR (95%CI)	Routine OGTT^#^ (n = 313)	Early OGTT^#^ (n = 170)	P value	Model 1^&^ Unadjusted OR (95%CI)	Model 2^&^ Adjusted OR (95%CI)
**Primary outcomes**										
LGA (>10^th^percentile)	118 (15.9%)	33 (18.8%)	0.36	1.22 (0.80-1.87)	1.13 (0.73-1.75)	47 (15.0%)	30 (17.6%)	0.45	1.21 (0.73-2.00)	1.17 (0.70-1.96)
**Secondary outcomes**										
Primary cesarean delivery	285 (38.5%)	70 (39.8%)	0.75	1.06 (0.76-1.48)	0.99 (0.71-1.40)	126 (40.3%)	66 (38.8%)	0.94	0.94 (0.64-1.38)	0.93 (0.63-1.37)
SGA (<10^th^percentile)	36 (4.9%)	5 (2.8%)	0.24	0.57 (0.22-1.48)	0.56 (0.22-1.47)	17 (5.4%)	5 (2.9%)	0.21	0.53 (0.19-1.46)	0.53 (0.19-1.48)
Preterm birth (28-37gw)	45 (6.1%)	16 (9.1)	0.15	1.55 (0.85-2.81)	1.52 (0.83-2.79)	23 (7.3%)	15 (8.8%)	0.57	1.22 (0.62-2.41)	1.25 (0.63-2.49)
Shoulder dystocia or forceps	23 (3.1%)	10 (5.7%)	0.10	1.88 (0.88-4.03)	1.92 (0.89-4.14)	8 (2.6%)	10 (5.9%)	0.07	2.38 (0.92-6.16)	2.35 (0.90-6.13)
Preeclampsia	18 (2.4%)	8 (4.5%)	0.13	1.91 (0.82-4.47)	1.64 (0.69-3.92)	7 (2.2%)	6 (3.5%)	0.40	1.60 (0.53-4.84)	1.62 (0.53-4.99)
Neonatal hypoglycemia (<2.2mmol/L)	3 (0.4%)	2 (1.1%)	0.24	2.83 (0.47-17.05)	3.15 (0.52-19.13)	2 (0.6%)	2 (1.2%)	0.53	1.85 (0.26-13.26)	1.94 (0.27-14.00)
Hyperbilirubinemia	28 (3.8%)	18 (10.2%)	<0.01	2.90 (1.57-5.38)	2.89 (1.55-5.37)	11 (3.5%)	18 (10.6%)	<0.01	3.25 (1.50-7.06)	3.17 (1.45-6.90)
Low Apgar (≤7)	15 (2.0%)	2 (1.1%)	0.43	0.56 (0.13-2.46)	0.49 (0.11-2.17)	9 (2.9%)	2 (1.2%)	0.23	0.40 (0.09-1.88)	0.39 (0.08-1.83)

^#^Data was shown by N (%).

^&^Data was shown by odds ratio (95%CI).

Model 1: unadjusted logistic regression model.

Model 2: adjusted for maternal age, BMI, HbAlc, smoking and family history of DM or previous GDM.

### *Post hoc* Analysis for Selective Outcomes by Subgroups According to maternal HbA1c Level, Age, BMI, Number of Parturitions and Neonatal Sex

**Figure 2:**
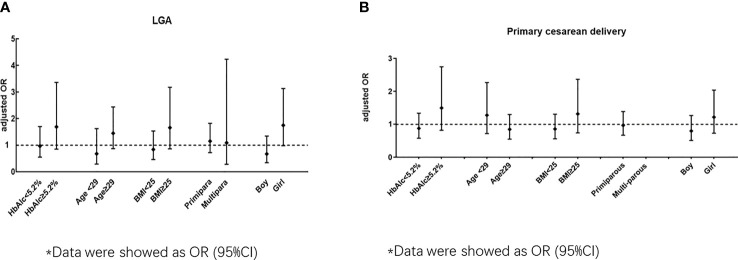
**(A)** Odds ratio (early OGTT/ routine OGTT) for LGA by subgroup according to HbAlc, age BMI, primipara and neonatal sex). **(B)** Odds ratio (early OGTT/ routine OGTT) for primary cesarean by subgroup according to HbAlc, age BMI, primipara and neonatal sex)*. *Data were showed as OR (95%CI).

The ORs stratified by maternal or neonatal factors for LGA between the early and routine OGTT groups are shown in **Figure 2A** and **Tables S2–S6**. In women who delivered a girl neonate, an early OGTT tended to be associated with an increased risk of LGA (unadjusted OR 1.84, 95% CI 1.04-3.27), but this trend became nonsignificant in the adjusted model (P>0.05) (**Table S6**). In other stratified models, including maternal HbA1c level, age, BMI and number of parturitions, no significant association was presented for LGA (adjusted models, P>0.05) (**Figure 2A**, and **Tables S3–S6**).

The ORs stratified by maternal or neonatal factors for primary cesarean section between the early and routine OGTT groups are shown in **Figure 2B** and **Tables S2–S6**. In all the stratified models for maternal HbA1c level, age, BMI, number of parturitions and neonatal sex, no significant association was observed for primary cesarean section between the early OGTT and routine OGTT groups (adjusted models, P>0.05) (**Figure 2B** and **Tables S2–S6**).

### Adherence

43 women in the early OGTT group who needed a second OGTT but did not receive it were regarded as lost to follow-up, as some of them may have had GDM and received therapy. Thus, adherence was > 95% (95.31%) in 917 participants. A sensitivity analysis taking into account adherence to intervention is shown in **Table S1**.

## Discussion

### Main Finding and Significance

Our results did not support that an early OGTT among women with intermediate hyperglycemia at the first prenatal visit improved pregnancy outcomes. An early OGTT did not reduce the risk of delivering LGA neonates but increased the risk of neonatal hyperbilirubinemia. There was no evidence of any difference between the groups in the occurrence of primary cesarean birth, preterm birth, shoulder dystocia or forceps delivery, maternal preeclampsia, neonatal hypoglycemia, or low Apgar score. Adherence to intervention was good, with an estimated >95% of the participants following the medical advice. To the best of our knowledge, this is the first study focusing on the effects of early screening for GDM among women with intermediate hyperglycemia at the first prenatal visit in a real-world setting. Our results suggested that among women with intermediate hyperglycemia at the first prenatal visit, an early OGTT did not improve pregnancy outcomes, including LGA.

### Comparison With Previous Studies

In the present study, an early OGTT during the first-half pregnancy was performed among women with intermediate hyperglycemia at the first prenatal visit and we did not find it to improve pregnancy outcomes. Our findings are consistent with previous studies, which evaluated the value of an early OGTT in other high-risk women or unselected pregnancies (9, 15–17). In contrast, there have been reports of the effects of an early OGTT on a reduced (5, 18, 19) or increased risk of LGA (20–23)among women with or without other high-risk factors. Different backgrounds of study populations may explain the differences in the results. This was the first study to evaluate the effects of an early OGTT during the first-half pregnancy versus a routine OGTT after 24 gw among women with intermediate hyperglycemia at the first prenatal visit. Consequently, there was no benefit of an early OGTT when the time of screening was initiated as early as the first-half pregnancy. Indeed, our findings that the incidence of neonatal hyperbilirubinemia is increased in women with an early OGTT suggest that such an intervention may be harmful. This adverse effect may be associated with fetal hyperinsulinemia. However, further study is warranted, as the pathogenesis of neonatal hyperbilirubinemia is complicated. Additionally, in women who later delivered a female baby, an early OGTT increased the risk of LGA, indicating the harm of this intervention among mothers with a female baby. However, the results of *post hoc* analyses should be considered exploratory, and further evidence is needed to demonstrate this observation. Overall, the results of our study suggest that women with intermediate hyperglycemia at the first prenatal visit do not benefit from an early OGTT.

### Interpretation

Intermediate hyperglycemia at the first prenatal visit has been reported to increase the risk of GDM, diagnosed after 24 gw, by approximately ten times, contributing to a higher frequency of LGA and primary cesarean section (7). Similarly, in the present study, the occurrence of women with LGA in all participants, irrespective of interventions of an early or a routine OGTT, was as high as 16.45%, comparable to that reported in women with GDM (2). In this respect, women with intermediate hyperglycemia could be regarded and treated as those with GDM directly, supporting the previous IADPSG recommendation of diagnosis of GDM at an FPG≥5.1 mmol/L at early pregnancy (2). However, in our present study, interventions of an early OGTT and standard management of glycemia control did not reduce the risk of LGA or other pregnancy outcomes. Thus, our results did not support the recommendation of the IAPDSG of classifying an FPG≥5.1 mM as GDM before 24 gw. The reason for our negative results may be that the GDM diagnostic criteria used at 24-28 gw may not be appropriate to identify women who truly need interventions, as intermediate hyperglycemia may involve a heterogeneous population. Hyperglycemia in early pregnancy could be related only to maternal metabolic disturbance due to a specific health status, such concomitant abnormal lipid metabolism (24) or maternal obesity (25). Additionally, it should be noted that all participants received lifestyle education in this real-world study, which may also contribute partly to this negative outcome. Overall, women with intermediate hyperglycemia are undoubtedly at high risk for GDM and adverse pregnancy. An early OGTT may not be effective in improving pregnancy outcomes among women with intermediate hyperglycemia in real world setting. Further data are needed to identify the truly high-risk pregnancies among these heterogeneous population.

### Strengths and Limitations

There were several strengths of the present study. First, some important variables, such as maternal age and BMI, were distributed uniformly between the early screening and routine screening groups and were included in various regression models to minimize the effects of potential confounding factors. Second, our study enrolled only women with an FPG greater than or equal to 5.1 mM and less than 7.0 mM, and women with preexisting GDM were excluded to maximize the ability to distinguish preexisting undiagnosed diabetes from GDM. Third, this was a single-center study conducted in one of the most famous tertiary obstetric hospitals in China. All the patients were treated by the same medical team using a standardized protocol based on the IADPSG guidelines to minimize unnecessary treatments.

However, there were also some limitations to this retrospective study. First, there were no further data on maternal weight gain, use of insulin and concentrations of HbA1c before delivery to demonstrate the effects of GDM management. Furthermore, as all the participants were educated regarding the adverse effects of GDM and encouraged to adopt a healthy diet and lifestyle habits, the possibility that the negative findings of an early OGTT on pregnancy outcomes were due to the women receiving extra advice could not be excluded. However, it had advantage, because in real world, these women are at high risk of further GDM and lifestyle education should be provided; thus, we provided evidence that at least in real world, women with intermediate hyperglycemia could not benefit from early OGTT, and further prospective study could be needed to develop the optimal managements for these women. Third, only infants with preterm birth, SGA, LGA, macrosomia, and low birth weight who were born to mothers with GDM underwent capillary blood tests, resulting in underestimation of neonatal hypoglycemia. Although there were no statistically significant associations between an early OGTT and most of the primary and secondary pregnancy outcomes, the effects on neonatal hypoglycemia need further estimation.

## Conclusions

This is the first study to evaluate the effects of an early OGTT among women with intermediate hyperglycemia at the first pregnancy visit. Women with intermediate hyperglycemia have a high occurrence of LGA and should be regarded as high-risk for GDM. In the present real-world study, early OGTT, accompanied with routine lifestyle education, did not improve pregnancy outcomes. Our results indicated that GDM diagnostic criteria used at 24-28 gw may not be appropriate to identify women who truly need interventions, and further new diagnostic criteria are needed.

## Data Availability Statement

All datasets analysed during the current study are not publicly available but are available from the corresponding authors on reasonable request.

## Author Contributions

Conception or design: XL and XX. Acquisition, analysis, or interpretation of data: YY, YX, and JW. Drafting the work or revising: YY and QZ. Final approval of the manuscript: XL and XX. All authors contributed to the article and approved the submitted version.

## Funding

This work was supported by grants from Shanghai Sailing Program (20YF1403100), Shanghai Key Program of Clinical Science and Technology Innovation (17411950500, 18511105602, 17411950501), Shanghai Medical Center of Key Programs for Female Reproductive Diseases (2017ZZ01016), National Natural Science Foundation of China (81871183, 8197061089), National Natural Science Foundation for Young Scholars of China (81701470), Shanghai Committee of Science and Technology (18411963400).

## Conflict of Interest

The authors declare that the research was conducted in the absence of any commercial or financial relationships that could be construed as a potential conflict of interest.

## Publisher’s Note

All claims expressed in this article are solely those of the authors and do not necessarily represent those of their affiliated organizations, or those of the publisher, the editors and the reviewers. Any product that may be evaluated in this article, or claim that may be made by its manufacturer, is not guaranteed or endorsed by the publisher.
