# Supplementary material for: Early Versus Routine Oral Glucose Tolerance Test in Women With Intermediate Hyperglycemia at First Prenatal Visit: A Retrospective Cohort Study in China
Source: Front Endocrinol (Lausanne). 2021 Dec 15;12:743170. doi: 10.3389/fendo.2021.743170 (PMC8716312; doi:10.3389/fendo.2021.743170)
Supplement: Supplementary file 1 [file Table_1.pdf]

Table S1. Primary and secondary outcomes between early OGTT and routine OGTT excluding cases that did not undergo a second OGTT*.

	Before Propensity-score matching					After Propensity-score matching				
	Routine OGTT (n=741)	Early OGTT (n=133)	P value	Model 1 Unadjusted OR (95%CI)	Model 2 Adjusted OR (95%CI)	Routine OGTT (n=313)	Early OGTT (n=128)	P value	Model 1 Unadjusted OR (95%CI)	Model 2 Adjusted OR (95%CI)
Primary outcomes										
LGA (>10thpercentile)	118(15.9%)	25(18.8%)	0.41	1.22(0.76-1.97)	1.12(0.69-1.81)	47(15.0%)	23(18.0%)	0.44	1.24(0.72-2.14)	1.16(0.66-2.04)
Secondary outcomes										
Primary cesarean delivery	285(38.5%)	51(38.3%)	0.98	1.0(0.68-1.46)	0.91(0.62-1.34)	126(40.3%)	47(36.7%)	0.49	0.86(0.56-1.32)	0.84(0.54-1.28)
SGA (<10thpercentile)	36(4.9%)	2(1.5%)	0.08	0.30(0.07-1.26)	0.56(0.22-1.47)	17(5.4%)	2(1.6%)	0.07	0.28(0.06-1.21)	0.53(0.19-1.48)
Preterm birth (28-37gw)	45(6.1%)	11(8.3%)	0.34	1.40(0.70-2.77)	1.35(0.67-2.71)	23(7.3%)	10(7.8%)	0.87	1.07(0.49-2.31)	1.09(0.50-2.38)
Shoulder dystocia or forceps	23(3.1%)	9(6.8%)	0.04	2.27(1.02-5.01)	2.28(1.02-5.09)	8(2.6%)	9(7.0%)	0.03	2.88(1.09-7.65)	2.75(1.03-7.40)
Preeclampsia	18(2.4%)	6(4.5%)	0.18	1.90(0.74-4.87)	1.48(0.56-3.94)	7(2.2%)	4(3.1%)	0.59	1.41(0.41-4.90)	1.35(0.38-4.78)
Neonatal hypoglycemia (<2.2mmol/L)	3(0.4%)	1(0.8%)	0.59	1.86(0.19-18.05)	2.17(0.22-21.26)	2(0.6%)	1(0.8%)	0.87	1.22(0.11-13.62)	1.38(0.12-15.58)
Hyperbilirubinemia	28(3.8%)	14(10.5%)	<0.01	3.00(1.53-5.86)	3.02(1.54-5.95)	11(3.5%)	14(10.9%)	<0.01	3.37(1.49-7.64)	3.31(1.45-7.56)
Low Apgar（≤7）	15(2.0%)	2(1.5%)	0.69	0.74(0.17-3.27)	0.62(0.14-2.78)	9(2.9%)	2(1.6%)	0.42	0.54(0.11-2.52)	0.49(0.10-2.32)

*Exclusion of these without second OGTT (N=43) in the early OGTT group.

Data was shown by N (%).

& Data was shown by odds ratio (95%CI).

Model 1: unadjusted logistic regression model.

Model 2: adjusted for maternal age, BMI, HbAlc, smoking and family history of DM or previous GDM.

Table S2 odd ratios (early OGTT/routine OGTT) for two selective outcomes measures by subgroup according to HbAlc.

Before Propensity-Score Matching						After Propensity-Score Matching				
	Routine OGTT (n=741)	Early OGTT (n=176)	P value	Model 1 Unadjusted OR (95%CI)	Model 2 Adjusted OR (95%CI)	Routine OGTT (n=313)	Early OGTT (n=170)	P value	Model 1 Unadjusted OR (95%CI)	Model 2 Adjusted OR (95%CI)
HbAlc<5.2%*	N=489	N=124		/	/	N=193	N=124		/	/
LGA (>10th percentile)	71(14.5%)	18(14.5)	0.10	1.0(0.57-1.75)	0.97(0.55-1.70)	22(11.4%)	18(14.5%)	0.42	1.32(0.68-2.58)	1.43(0.72-2.85)
Primary cesarean delivery	174(35.6%)	42(33.9%)	0.72	0.93(0.61-1.41)	0.88(0.58-1.34)	76(39.4%)	42(33.9%)	0.32	0.79(0.49-1.26)	0.80(0.49-1.28)
HbAlc≥5.2%*	N=252	N=52		/	/	N=120	N=46		/	/
LGA (>10th percentile)	47(18.7%)	15(28.8%)	0.10	1.77(0.90-3.49)	1.69(0.85-3.36)	25(20.8%)	12(26.1%)	0.47	1.34(0.61-2.96)	1.21(0.53-2.73)
Primary cesarean delivery	111(44.0%)	28(53.8%)	0.20	1.48(0.81-2.70)	1.50(0.82-2.75)	50(41.7%)	24(52.2%)	0.22	1.53(0.77-3.02)	1.66(0.81-3.37)

*Cut-off of HbAlc was made at 5.5% according to 90%th of general population in recent study.

Data was shown by N (%).

& Data was shown by odds ratio (95%CI).

Model 1: unadjusted logistic regression model.

Model 2: adjusted for maternal age, BMI, smoking, and family history of DM or previous GDM.

TableS3. odd ratios (early OGTT/routine OGTT) for two selective outcomes measures by subgroup according to maternal age.

Before Propensity-Score Matching						After Propensity-Score Matching				
	Routine OGTT (n=741)	Early OGTT (n=176)	P value	Model 1 Unadjusted OR (95%CI)	Model 2 Adjusted OR (95%CI)	Routine OGTT (n=313)	Early OGTT (n=170)	P value	Model 1 Unadjusted OR (95%CI)	Model 2 Adjusted OR (95%CI)
Age <29*	N=311	N=62		/	/	N=122	N=61		/	/
LGA (>10th percentile)	50(16.1%)	7(11.3%)	0.34	0.67(0.29-1.54)	0.68(0.29-1.62)	20(16.4%)	7(11.5%)	0.38	0.66(0.26-1.66)	0.75(0.28-2.05)
Primary cesarean delivery	105(33.8%)	25(40.3%)	0.32	1.32(0.76-2.32)	1.28(0.72-2.27)	43(35.2%)	24(39.3%)	0.59	1.19(0.63-2.25)	1.21(0.63-2.32)
Age≥29*	N=430	N=114		/	/	N=191	N=109		/	/
LGA (>10th percentile)	68(15.8%)	26(22.8%)	0.08	1.57(0.95-2.62)	1.45(0.87-2.44)	27(14.1%)	23(21.1%)	0.06	1.62(0.88-3.00)	1.60(0.85-3.02)
Primary cesarean delivery	180(41.9%)	45(39.5%)	0.64	0.91(0.59-1.38)	0.85(0.55-1.30)	83(43.5%)	42(38.5%)	0.41	0.82(0.51-1.32)	0.76(0.47-1.25)

*Cut-off of maternal age was made at 29 according to our previous finding of optimized cutoff maternal age for GDM.

Data was shown by N (%).

& Data was shown by odds ratio (95%CI).

Model 1: unadjusted logistic regression model.

Model 2: adjusted for BMI, HbAlc, smoking and family history of DM or previous GDM.

TableS4. odd ratios (early OGTT/routine OGTT) for two selective outcomes measures by subgroup according to maternal BMI.

Before Propensity-Score Matching						After Propensity-Score Matching				
	Routine OGTT (n=741)	Early OGTT (n=176)	P value	Model 1 Unadjusted OR (95%CI)	Model 2 Adjusted OR (95%CI)	Routine OGTT (n=313)	Early OGTT (n=170)	P value	Model 1 Unadjusted OR (95%CI)	Model 2 Adjusted OR (95%CI)
BMI<25*	N=534	N=117		/	/	N=229	N=116		/	/
LGA (>10thpercentile)	73(13.7%)	15(12.8%)	0.81	0.93(0.51-1.69)	0.84(0.46-1.53)	28(12.2%)	15(12.9%)	0.85	1.07(0.55-2.09)	1.09(0.55-2.18)
Primary cesarean delivery	191(35.8%)	39(33.3%)	0.62	0.90(0.59-1.37)	0.86(0.56-1.31)	87(38.0%)	39(33.6%)	0.43	0.83(0.52-1.32)	0.87(0.54-1.39)
BMI⩾25*	N=207	N=59		/	/	N=84	N=54		/	/
LGA (>10thpercentile)	45(21.7%)	18(30.5%)	0.16	1.58(0.83-3.01)	1.66(0.86-3.18)	19(22.6%)	15(27.8%)	0.49	1.32(0.60-2.88)	1.38(0.62-3.06)
Primary cesarean delivery	94(45.4%)	31(52.5%)	0.33	1.33(0.75-1.15)	1.32(0.74-2.37)	39(46.4%)	27(50.0%)	0.68	1.15(0.58-2.29)	1.15(0.56-2.34)

*cut-off of maternal BMI was made at 29 according to definition of overweight or not.

Data was shown by N (%).

& Data was shown by odds ratio (95%CI).

Model 1: unadjusted logistic regression model.

Model 2: adjusted for maternal age, HbAlc, smoking and family history of DM or previous GDM.

Table S5. odd ratios (early OGTT/routine OGTT) for two selective outcomes measures by subgroup according to number of parturitions.

	Before Propensity-Score Matching					After Propensity-Score Matching				
	Routine OGTT (n=741)	Early OGTT (n=176)	P value	Model 1 Unadjusted OR (95%CI)	Model 2 Adjusted OR (95%CI)	Routine OGTT (n=313)	Early OGTT (n=170)	P value	Model 1 Unadjusted OR (95%CI)	Model 2 Adjusted OR (95%CI)
Primiparous	N=651	N=156				N=276	N=152			
LGA (>10thpercentile)	101(15.5%)	29(18.6%)	0.35	1.24(0.79-1.96)	1.15(0.72-1.82)	42(15.2%)	27(17.8%)	0.49	1.20(0.71-2.05)	1.19(0.69-2.05)
Primary cesarean delivery	285(43.8%)	70(44.9%)	0.81	1.05(0.74-1.49)	0.97(0.67-1.39)	126(45.7%)	66(43.4%)	0.66	0.91(0.61-1.36)	0.93(0.62-1.40)
Multi-parous	N=90	N=20				N=37	N=18			
LGA (>10thpercentile)	17(18.9%)	4(20.0%)	0.91	1.07(0.32-3.62)	1.09(0.28-4.23)	5(13.5%)	3(16.7%)	0.76	1.28(0.27-6.07)	1.14(0.18-7.44)
Primary cesarean delivery	0	0	/	/	/	0	0	/	/	/

Data was shown by N (%).

& Data was shown by odds ratio (95%CI).

Model 1: unadjusted logistic regression model.

Model 2: adjusted for maternal age, BMI, HbAlc, smoking and family history of DM or previous GDM.

Table S6. odd ratios (early OGTT/routine OGTT) for two selective outcomes measures by subgroup according to neonatal sex.

	Before Propensity-Score Matching					After Propensity-Score Matching				
	Routine OGTT (n=741)	Early OGTT (n=176)	P value	Model 1 Unadjusted OR (95%CI)	Model 2 Adjusted OR (95%CI)	Routine OGTT (n=313)	Early OGTT (n=170)	P value	Model 1 Unadjusted OR (95%CI)	Model 2 Adjusted OR (95%CI)
Boy	N=386	N=99		/	/	N=156	N=97		/	/
LGA (>10th percentile)	58(15.0%)	12(12.1%)	0.46	0.78(0.40-1.52)	0.67(0.34-1.34)	23(14.7%)	11(11.3%)	0.44	0.74(0.34-1.59)	0.65(0.30-1.45)
Primary cesarean delivery	165(42.7%)	39(39.4%)	0.55	0.87(0.56-1.37)	0.80(0.51-1.27)	70(44.9%)	37(38.1%)	0.29	0.76(0.45-1.27)	0.75(0.45-1.27)
Girl	N=355	N=77		/	/	N=157	N=73		/	/
LGA (>10th percentile)	60(16.9%)	21(27.3%)	0.04	1.84(1.04-3.27)	1.75(0.98-3.13)	24(15.3%)	19(26.0%)	0.05	1.95(0.99-3.85)	2.26(1.10-4.60)
Primary cesarean delivery	120(33.8%)	31(40.3%)	0.28	1.32(0.80-2.19)	1.22(0.73-2.04)	56(35.7%)	29(39.7%)	0.55	1.19(0.67-2.11)	1.12(0.62-2.03)

Data was shown by N (%).

& Data was shown by odds ratio (95%CI).

Model 1: unadjusted logistic regression model.

Model 2: adjusted for maternal age, BMI, HbAlc, smoking and family history of DM or previous GDM.
